# *Pseudolebinthus lunipterus* sp. nov.: a striking deaf and mute new cricket from Malawi (Orthoptera, Gryllidae, Eneopterinae)

**DOI:** 10.7717/peerj.8204

**Published:** 2020-01-21

**Authors:** Karen Salazar, Raymond J. Murphy, Marion Guillaume, Romain Nattier, Tony Robillard

**Affiliations:** 1 Institut de Systématique, Evolution, Biodiversité (ISYEB), Muséum national d’Histoire naturelle, CNRS, Sorbonne Université, EPHE, Université des Antilles, Paris, France; 2 Grupo de Investigación Insectos de Colombia, Instituto de Ciencias Naturales, Universidad Nacional de Colombia, Bogotá, Colombia; 3 Unaffiliated, Mzuzu, Malawi

**Keywords:** New species, Acoustic communication, Malawi, Africa, Xenogryllini, Mitogenome, Molecular phylogeny, Cricket, Evolution of communication, Taxonomy

## Abstract

This article presents an intriguing new cricket species of the tribe Xenogryllini discovered in Northern Malawi. This is the first case of mute and deaf species in the subfamily Eneopterinae; it shows no stridulatory apparatus on short male forewings and no tympana on either side of fore tibiae in both sexes. We introduce the new species and its complete mitogenome and assess phylogenetic relationships based on molecular data obtained from next-generation sequencing genome skimming method. Phylogenetic analyses place the new species within the genus *Pseudolebinthus* in Xenogryllini, as the sister species of *Pseudolebinthus gorochovi* Robillard. We describe *Pseudolebinthus lunipterus* sp. nov., provide illustrations of main morphology, male and female genitalia, photographs of living specimens and information about habitat and update the identification key for species of genus *Pseudolebinthus*. We discuss the differences between the new species and related taxa and the striking loss of acoustic communication in this cricket.

## Introduction

Crickets have long been studied for their capacity to communicate with sound (Horch et al., 2017). Their mechanism of sound production by wing stridulation and their hearing system has been extensively studied during the last 50 years (Michelsen, 1998; Bennet-Clark, 1989; Gerhardt & Huber, 2002). What is less known is that many lineages within the cricket clade have independently lost their capacity to produce sound, and sometimes their hearing capacity too. Numerous examples of mute lineages are spread in the taxonomic literature about crickets (Otte & Alexander, 1983; Otte, 1992; Wang et al., 2018), some species being completely wingless and some retaining the wings while losing stridulatory structures at different degrees (Zuk, Rotenberry & Tinghitella, 2006; Pascoal et al., 2014). Recent studies have demonstrated that the loss of sound production structures on male forewings (FWs) could occur convergently and very rapidly in populations of *Teleogryllus oceanicus* (Le Guillou) as a result to strong selective pressures by a parasitoid fly (Zuk, Rotenberry & Tinghitella, 2006; Pascoal et al., 2014).

In many mute lineages of crickets, auditory tympana are retained after the tegminal stridulatory mechanism is lost (Otte, 1992), which could be linked with avoidance of bat predation. Species still able to fly but in which males have lost the stridulum usually retain the tympana (Otte & Alexander, 1983; Otte, Alexander & Cade, 1987). Species becoming both mute and deaf are relatively less common, even among the diversity of situations presented by crickets.

The subfamily Eneopterinae has been well studied for its diversity of traits related to acoustic communication (Robillard & Desutter-Grandcolas, 2004a, 2004b; Robillard, Grandcolas & Desutter-Grandcolas, 2007; ter Hofstede et al., 2015). Among this diversity, eneopterines include several lineages which have lost capacity to produce sound independently (Robillard & Desutter-Grandcolas, 2004a), either by losing stridulatory structures while retaining long FWs (genus *Swezwilderia* Chopard, 1929 in tribe Lebinthini), or by becoming apterous (genus *Paranisitra* Chopard, 1925 in tribe Nisitrini), but no example of complete loss of hearing was documented yet.

**Figure 1:**
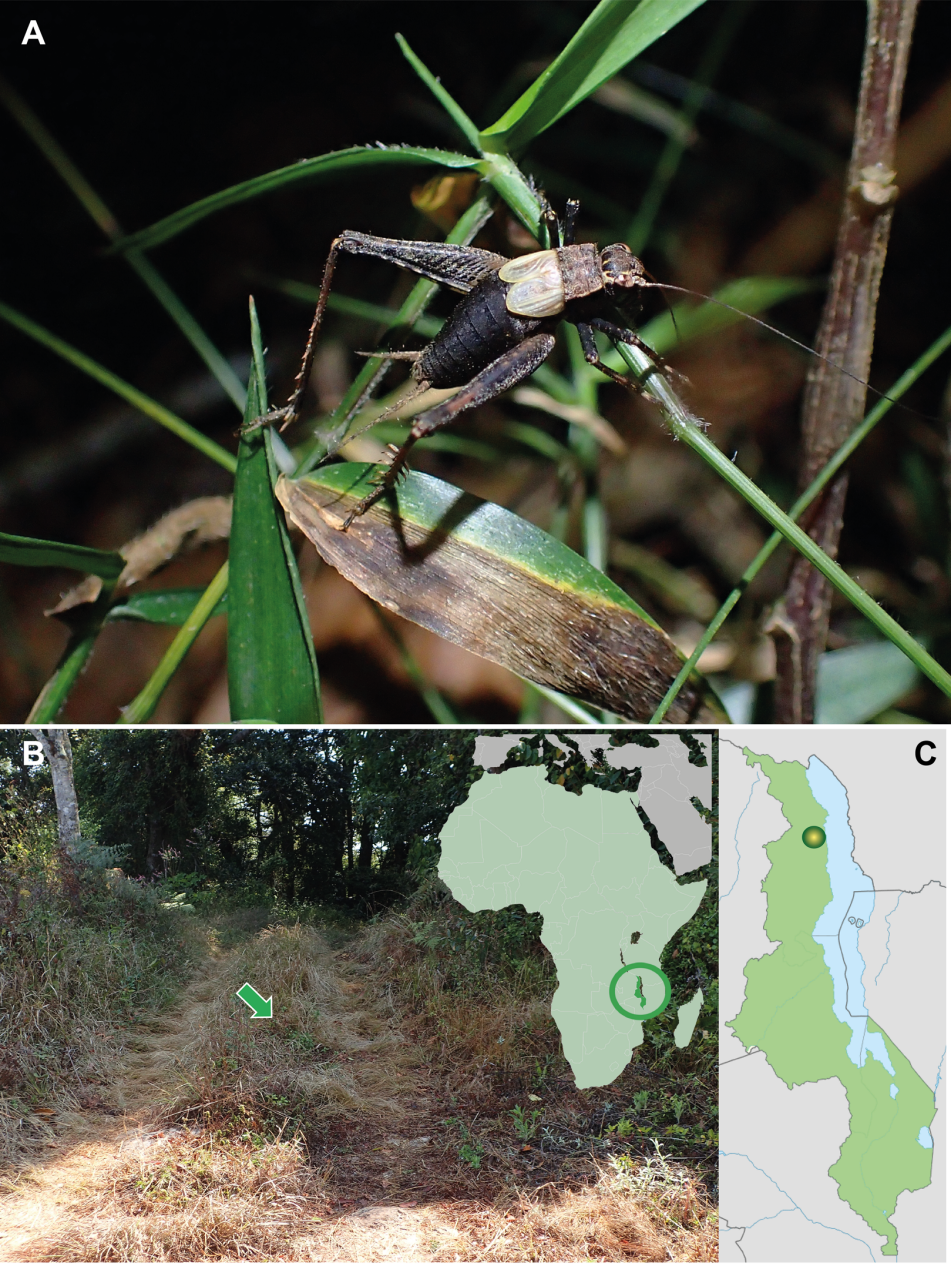
*Pseudolebinthus lunipterus* sp. nov. (A) Male habitus on low vegetation at night; (B) natural habitat indicated by a green arrow (left) and location of Malawi on simplified map of Africa (left); (C) type locality in Malawi. Photo Tony Robillard.

In this study, we describe the species *Pseudolebinthus lunipterus* sp. nov., a new eneopterine from Northern Malawi being both mute and deaf (Fig. 1). The new species is the first member of Eneopterinae showing no stridulatory apparatus on short male FWs and no tympana on either side of fore tibiae in both sexes. We provide illustrations about main morphology, male and female genitalia, photographs of living specimens and information about its natural habitat. We describe its complete mitogenome and assess phylogenetic relationships based on molecular data obtained by next-generation sequencing using the genome skimming method (Straub et al., 2012). We discuss the differences between the new species and related taxa, their phylogenetic relationships and the possible origins of muteness and deafness of these crickets.

## Materials and Methods

### Material and taxonomy

The new collected material comes from a field expedition in Malawi in September and October 2018 under the collection and export permit number EAD-12-07-087-18-20a from The Forestry Research Institute of Malawi (FRIM) and from the personal collection of R.J.M. Specimens are deposited in the collections of Muséum national d’Histoire naturelle, Paris (MNHN).

The electronic version of this article in Portable Document Format will represent a published work according to the International Commission on Zoological Nomenclature (ICZN), and hence the new names contained in the electronic version are effectively published under that Code from the electronic edition alone. This published work and the nomenclatural acts it contains have been registered in ZooBank, the online registration system for the ICZN. The ZooBank Life Science Identifiers (LSIDs) can be resolved and the associated information viewed through any standard web browser by appending the LSID to the prefix http://zoobank.org/. The LSID for this publication is: urn:lsid:zoobank.org:pub:DA17C29A-B265-4819-A9E6-55FCB137E3F0. The online version of this work is archived and available from the following digital repositories: PeerJ, PubMed Central and CLOCKSS.

Description follows terminologies as proposed by Robillard et al. (2014). Observations of external morphological characters and dissection of male and female genitalia were performed using a Leica stereomicroscopes MZ16. Terminologies for male FW venation follow Ragge (1955) and Robillard & Desutter-Grandcolas (2004a). Male and female genitalia were dissected from freshly killed specimens. Male genitalia were dissected by making a small slit between paraproct and subgenital plate. Female copulatory papilla was dissected out by cutting the membrane between ovipositor and subgenital plate. Dissected genitalia were cleared in 10% cold KOH solution and preserved in glass vials containing glycerine. Imaging of male and female genitalia were made using Canon EOS 60D Digital SLR camera on a Nikon stereomicroscope SMZ1500. To highlight the structural components of male and female genitalia, water solution containing a drop of JBL Punktol was used. To fix orientation and stabilization of genitalia for photography, a clear and viscous Power Plast Hand Sanitizer was used following Su (2016). The dry mounted adults were photographed with a Canon EOS 6D Digital SLR camera.

### Abbreviations used in taxonomic descriptions and figures

General morphology: FI, FII, FIII, fore, median, hind femur; FW, forewing; TI, TII, TIII, fore, median, hind tibia; Tarsomere I/II/III-1: basal segment of fore, median and hind leg tarsomere. Tegminal venation: 1A–4A, first to fourth anal veins; CuA, anterior cubitus; CuP, posterior cubitus; M, median vein; R, radial vein; Sc, subcostal vein and its projections.

Male genitalia: ac gl, accessory gland; ect ar, ectophallic arc; ect ex, ectophallic lateral expansion; ect f, ectophallic fold; ect f s, sclerite of ectophallic fold; ect ap, ectophallic apodeme; end sc, endophallic sclerite; end ap, endophallic apodeme; pse, pseudepiphallus; pse pe, pseudepiphallic posterior expansion; pse lo, pseudepiphallic lophi; pse pa, pseudepiphallic paramere; pse ra, pseudepiphallic rami; pse re, pseudepiphallic basal reinforcement.

Measurements: (in mm, except for spine numbers) BL, body length in dorsal view, from fastigium to apex of abdomen; FIIIL, length of FIII; FIIIW, width of FIII; TIIIL, length of TIII; FWL, forewing length; FWW, forewing width (at the level of maximal width at about 1/3 of FWL); Ias, inner spines on TIII dorsal side above the spurs; Ibs, inner spines on TIII dorsal side between the spurs; Oas, outer spines on TIII dorsal side above the spurs; Obs, outer spines on TIII dorsal side between the spurs; OL, ovipositor length; PronL, pronotum length; PronW, pronotum width; TaIIIs, spines of third hind tarsomere, not including the apical spines: Ids, inner dorsal spines; Ods, outer dorsal spines; Ols, outer spines on lateral side of TaIII.

### Laboratory methods

DNA extraction, PCR amplification and bank preparations were carried out at Service de Systématique Moléculaire of the MNHN. We extracted DNA from ethanol-preserved median legs for four newly collected specimens (two *P. lunipterus* sp. nov. and two *Pseudolebinthus gorochovi* Robillard, 2018) (see Table 1 for details about specimen vouchers). Total genomic DNA was extracted using a DNeasy Blood and Tisue Kit (Qiagen Inc., Venlo, Netherlands and Germany) following the manufacturer’s instructions. For each newly generated extract, we amplified the mitochondrial gene maker 12S (*12S rRNA* gene, amplicon ~400 bp) with the protocols described in Nattier et al. (2012) with the following primers and annealing temperatures: 12SF 5′-TACTATGTTACGACTTAT′3′, 12sr 5′-AAACTAGGATTAGATACCC-3′ at 48 °C (Kambhampati, 1995). PCR products were sequenced with the Ion Torrent PGM platform in MNHN. Assembling and annotations were executed with Geneious Prime 2019.1.3 (Biomatter Ltd., Auckland, New Zealand, Oceania, www.geneious.com, Kearse et al., 2012).

**Table 1:** Taxon and gene sampling. List of all Eneopterinae crickets and outgroups used in this study with GenBank Accession Numbers. NA means missing data. Names and voucher codes of taxa sequenced in this study are in bold.

Species	Voucher/lab code (type status)	16S	12S	COI	COII	Cytb	18S	28S	H3
*Acheta domesticus* Linnaeus (1758)	MNHN-EO-ENSIF3523/Adom	AF248698	ADZ97611	JX897403	JX897439	AF248682	AD18SITS1	JX897465	KR903150
*Agnotecous meridionalis* Desutter-Grandcolas (2006)	MNHN-ENSIF-2772/AmeIP	JX897349	JX897401	JX897420	NA	JX897311	JX897579	JX897488	JX897553
*Agnotecous meridionalis* Desutter-Grandcolas (2006)	MNHN-EO-ENSIF-2771/AmePB	JX897350	JX897402	JX897410	JX897442	JX897313	JX897597	JX897489	JX897550
*Cardiodactylus novaeguineae* Haan (1844)	MNHN-ENSIF2038/C3CnoNC	JF972520	JF972504	MH662977	MH662880	JF972488	JF972535	MH663420	NA
*Cardiodactylus novaeguineae* Haan (1844)	MNHN-EO-ENSIF2030/C2CnoPe	JF972521	JF972506	KU705563	KU705551	JF972490	JF972537	KR903500	KR903151
*Eneoptera guyanensis* Chopard (1931)	MNHN-EO-ENSIF2741/Egu	AY905301	AY905272	JX897404	KU705553	AY905355	AY905331	KU705581	JX897547
Eurepini sp.	MNHN-EO-ENSIF3155/Eursp	KR903674	KR903834	KU705565	KU705554	KR903331	KR904028	KR903503	KR903153
*Gryllus bimaculatus* De Geer (1773)	MNHN-EO-ENSIF3524/3404/Gbi	AF248685	AY905292	NA	KU705555	AF248659	AF514509	KR903002	KR903154
*Indigryllus kudremu* sp. nov.	ZSI/X3Xsp2 (AT)	KY595509	KY595483	KY646248	MK761340	AY905377	AY905345	KY605247	KY646293
*Lebinthus bitaeniatus* Stål (1877)	MNHN-EO-ENSIF4393/L18LbiP1	MK761250	MK761274	MK761331	MK761341	MK761353	MK761293	MK761313	MK761370
*Lebinthus bitaeniatus* Stål (1877)	MNHN-EO-ENSIF4394/L28LbiP2	MK761252	MK761275	MK761332	NA	MK761354	MK761294	MK761314	MK761371
*Lebinthus luae* Robillard & Tan (2013)	MNHN-EO-ENSIF2740/L8LbiS1 (PT)	JF972524	KR904017	KU705567	KU705557	JF972493	KR904199	KR903665	KR903321
*Lebinthus luae* Robillard & Tan (2013)	MNHN/L10LbiS3	MK761253	MK761276	MK761333	MK761342	MK761355	MK761295	MK761315	MK761372
*Microbinthus santoensis* Robillard (2009)	MNHN-EO-ENSIF2484/L7LsaPe	KU705528	KU708011	KU705569	NA	KU705535	KU705543	KU705585	KU705601
*Microbinthus santoensis* Robillard (2009)	MNHN-EO-ENSIF2437/LsaV (PT)	JF972527	JF972511	JX897405	JX897441	JF972495	JF972542	JX897467	JX897548
*Nisitrus vittatus* Haan (1842)	MNHN-EO-ENSIF2742/NviS	MH575026	MH575158	KU705572	NA	MH662741	AY905340	KR903667	JX897546
*Pseudolebinthus gorochovi* Robillard (2018)	ZIN/X17PsMal1 (HT)	KY595508	KY595472	KY646231	NA	NA	KY595511	KY605231	MK761373
***Pseudolebinthus gorochovi* Robillard (2018)**	**MNHN-EO-ENSIF10732/X27**	NA	**MN583263**	NA	NA	NA	NA	NA	NA
***Pseudolebinthus gorochovi* Robillard (2018)**	**MNHN-EO-ENSIF10744/X31**	NA	**MN583262**	NA	NA	NA	NA	NA	NA
***Pseudolebinthus lunipterus* sp. nov.**	**MNHN-EO-ENSIF10720/X28 (PT)**	**MN414243**	**MN414243**	**MN414243**	**MN414243**	**MN414243**	**MN583259**	**MN583260**	**MN583264**
***Pseudolebinthus lunipterus* sp. nov.**	**MNHN-EO-ENSIF10718/X34 (PT)**	NA	**MN583261**	NA	NA	NA	NA	NA	NA
*Xenogryllus eneopteroides* Bolívar (1890)	MNHN-EO-ENSIF3159/XenAC	KR903829	KR904023	KY646249	NA	KR903490	KR904205	KR903670	KR903328
*Xenogryllus eneopteroides* Bolívar (1890)	MNHN-EO-ENSIF3442/XenCI	MK761256	MK761279	NA	NA	NA	MK761298	NA	MK761375
*Xenogryllus eneopteroides* Bolívar (1890)	MNHN-EO-ENSIF3442/XenGA	MK761257	MK761280	NA	NA	NA	MK761299	MK761318	MK761376
*Xenogryllus maichauensis* Gorochov (1992)	ZFMK/XtrTh	MK761258	NA	NA	NA	MK761357	NA	NA	MK761377
*Xenogryllus marmoratus* Haan (1844)	MNHN-EO-ENSIF3161/Xma2	KR903830	KR904024	NA	MK761343	KR903491	KR904206	NA	KR903329
*Xenogryllus marmoratus* Haan (1844)	MNHN-EO-ENSIF1599/XmaCh1	KY595510	KY595484	NA	MK761344	KY646274	KY595518	KY605248	KY646292
*Xenogryllus marmoratus* Haan (1844)	MNHN-EO-ENSIF1594/XmaCh2	MK761261	MK761283	NA	MK761345	MK761360	MK761302	MK761320	MK761379
*Xenogryllus marmoratus* Haan (1844)	MNHN-EO-ENSIF3562/XmaCh3	MK761262	MK761284	NA	MK761346	MK761361	MK761303	MK761321	MK761380
*Xenogryllus mozambicus* Robillard (2019)	MNHN-EO-ENSIF1515/XenMoz (PT)	MK761263	MK761285	MK761336	NA	MK761362	MK761304	NA	MK761381
*Xenogryllus transversus* Walker (1869)	IISERM/Xtr715	MK761264	MK761286	NA	NA	MK761363	MK761305	MK761322	MK761382
*Xenogryllus transversus* Walker (1869)	IISERM/Xtr765	MK761265	NA	MK761337	NA	MK761364	MK761306	MK761323	MK761383
*Xenogryllus transversus* Walker (1869)	IISERM/Xtr766	MK761266	NA	NA	NA	MK761365	NA	MK761324	MK761384
*Xenogryllus transversus* Walker (1869)	MNHN-EO-ENSIF87/XtrIn	JF972530	NA	KY646247	MK761347	JF972499	KY595519	KY605246	KY646294
*Xenogryllus ululiu* Gorochov (1990)	ZIN/X18XulV2	MK761268	MK761287	NA	MK761348	NA	MK761308	MK761326	MK761386
*Xenogryllus ululiu* Gorochov (1990)	ZIN/X19XulSi	MK761269	MK761288	NA	MK761349	MK761367	MK761309	MK761327	MK761387
*Xenogryllus ululiu* Gorochov (1990)	MNHN-EO-ENSIF4385/X20XulCam1	MK761270	MK761289	NA	MK761350	NA	MK761310	MK761328	MK761388
*Xenogryllus ululiu* Gorochov (1990)	ZIN/X21XulCam2	MK761271	MK761290	NA	MK761351	NA	MK761311	MK761329	MK761389
*Xenogryllus ululiu* Gorochov (1990)	ZFMK/Xulth	MK761272	MK761291	MK761339	NA	MK761368	NA	NA	NA

**Note:**

Abbreviation of museums: IISERM, Indian Institute of Science Education and Research Mohali, Punjab, India; MNHN, Muséum national d’Histoire naturelle, Paris, France; ZFMK, Zoologisches Forschungsinstitut und Museum Alexander Koenig, Bonn, Germany; ZIN, Zoological Institute, Russian Academy of Sciences, S. Petersburg, Russia, and ZSI, Zoological Survey of India, Kolkata, India. Other abbreviations: HT, Holotype; PT, Paratype.

One extract of the new species *P. lunipterus* (X28, MNHN-EO-ENSIF10720) was used for library preparation in a Genome Skimming approach (Straub et al., 2012). We assessed total DNA with a Qubit™ dsDNA High-Sensitivity Assay Kit (Life Technologies, Paisley, UK) with a Fluorescence Microplate Reader in 1.0 µL of sample. Prior to library preparation, we fragmented the DNA by sonication using BioRuptor^®^ UCD-200 (Life Technologies and Invitrogen, Carlsbad, CA, USA) using 50 µL DNA sample and 50 µL TE buffer 0.1X. The molecular weight of the fragmented DNA sample was analyzed in agarose gel electrophoreses (3.0 µL DNA sample plus BG 1.0 µL; gel agarose 1% in TAE buffer (Tris-acetate-EDTA) 1.0X; migration buffer TAE 0.5X; migration time 20 min) before and after the sonification of the DNA. We then used the NEBNext^®^ Ultra™ II DNA Library Prep Kit for Illumina (New England BioLabs, Ipswich, MA, USA; dsDNA protocol) with a modified version of the protocol based on Meyer & Kircher (2010). After library preparation, total DNA was quantified with a Qubit™ dsDNA (HS) Assay Kit using Qubit™ Fluorometer (Life Technologies, Carlsbad, CA, USA) in 1.0 µL of sample. Libraries were then analyzed with a Bioanalyzer 2100 DNA 1000 series II chip (Agilent Technologies, Santa Clara, CA, USA) (High Sensitivity DNA Assay). Pooled libraries were sequenced as paired-end reads (150 bp) on an Illumina HiSeq 3000 HWI-J0015 at the Genome and Transcriptome Platform of Toulouse (Genotoul, Toulouse, France).

### Sequence analyses and mitogenome annotation

Sequencing reads from both paired-end libraries were imported in Geneious Prime 2019.1.3, then filtered and trimmed by quality using the BBDuk plugin (minimum quality score of 30 and minimum length of reads of 30 bp). Quality and length distribution of the sequences were inspected using FastQC v. 0.11.8 (Andrews, 2010) under the open-source application Galaxy (http://galaxyproject.org/) (Afgan et al., 2018). We then extracted sequences of interest from the total read using the *Map to reference* option in Geneious (Custom sensibility, fine tuning: iterate up to 10 times; Maximum Mismatches Per Read 30). The Mitochondrial genomes of *Xenogryllus marmoratus* (Haan, 1842) (Ma, Zhang & Li, 2019; GenBank Accession MK033622) and *Cardiodactylus muiri* Otte, 2007 (Dong et al., 2017; GenBank Accession MG680938) were used as references. After removing the reference sequence from the resulting contig, a step of De Novo *assemble* (Sensibility: High sensibility/Medium) was performed in Geneious. The longest resultant contig (Number of reads 14,817; Sequence length 16,075 bp) was chosen as a seed and mapped with the filtered reads again (Custom sensibility, fine-tuning: iterate up to 25 times; max. Mismatches Per Read 10).

The consensus sequence (Threshold: Highest quality; Assign quality: Highest) was then circularized and annotated with Geneious with *X. marmoratus* as reference. Genome annotation based on sequence similarity was performed independently using Geneious and with MITOS (Bernt et al., 2013), available at http://mitos.bioinf.uni-leipzig.de/index.py using the invertebrate mitogenome genetic code.

A similar process was used to extract sequences of three nuclear genes: histone H3 (*H3*, ~330 bp) and the sequences of the non-protein-coding genes corresponding to nuclear small ribosomal subunit (*18S rRNA*, *18S*, ~650 bp) and of the nuclear large ribosomal subunit (*28S rRNA*, *28S*, ~400 bp). Nuclear genes were assembled using references from the species *P. gorochovi* from (Jaiswara, Dong & Robillard, 2019). All the newly generated sequences are available on GenBank (Table 1).

### Phylogenetic analysis

The cricket tribe Xenogryllini is composed of three genera and 13 valid species (including the new species): *Xenogryllus* Bolívar (eight species), *Pseudolebinthus* Robillard (four species) and *Indigryllus* Robillard & Jaiswara (one species). To infer the phylogenetic position of the new species, we refer to the recent molecular phylogeny of Xenogryllini (Jaiswara, Dong & Robillard, 2019) which included eight Xenogryllini species representing the three genera and eight species representing all four other tribes of the Eneopterinae subfamily, plus two more distant species belonging to the subfamily Gryllinae. We used DNA markers from eight genes, five from the mitochondrial and three from the nuclear genome based on Jaiswara, Dong & Robillard (2019) and previous studies (Robillard & Desutter-Grandcolas, 2006; Nattier et al., 2012). The mitochondrial markers were partial sequences of the small subunit *rRNA* gene (*12S*, amplicon ~400 bp), the large subunit *rRNA* gene (*16S*, ~500 bp), of the *cytochrome b* gene (*Cytb*, ~400 bp), and of the *cytochrome c oxidase subunit 1* (*CO1*, ~750 bp) and *subunit 2* (*CO2*, ~400 bp). Nuclear markers were partial sequences of protein coding *histone H3* gene (*H3*, ~330 bp), and partial sequences of two non-protein-coding genes corresponding to nuclear ribosomal subunits *18S rRNA* (*18S*, ~650 bp) and *28S rRNA* (*28S*, ~400 bp).

Newly generated sequences were added to the previous data set. We extracted the mitochondrial markers from the mitogenome of *P. lunipterus* sp. nov. See Table 1 for detailed information about taxon and molecular sampling.

The sequences were aligned with MAFFT version 7 online (Katoh & Standley, 2013). The aligned sequences of all eight markers were further concatenated in Sequencematrix (Vaidya, Lohman & Meier, 2011). The concatenated dataset was analyzed using maximum likelihood (ML) using IQ-TREE 1.6. 2 web portal (Nguyen et al., 2015) (http://iqtree.cibiv.univie.ac.at/; Kalyaanamoorthy et al., 2017; Trifinopoulos et al., 2016) with data partitioned by gene marker and the following options: Edge-unlinked partitions, Substitution model Auto. Clade support was assessed by conducting 1,000 bootstrap replicates (standard bootstrap). Nodes supported by bootstrap support values (BS) ≥ 70% were considered strongly supported.

## Results

### Mitogenome of *P. lunipterus*

**Figure 2:**
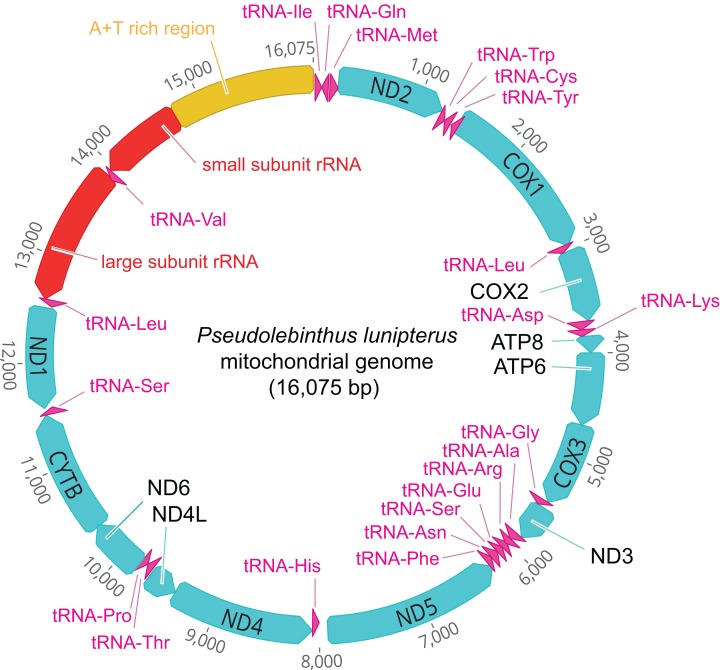
Map of the mitochondrial genome of *Pseudolebinthus lunipterus* sp. nov. The 13 PCGs are shown in blue, the 22 tRNA in purple, the 2 *rRNA* genes in red and the A + T rich region in orange. The direction of transcription is indicated by an arrow.

The mitogenome of *P. lunipterus* is 16,075 bp in length and has a typical circular structure (Fig. 2). The nucleotide composition of this genome has a GC content of 24.2%. The identity and position of 13 PCGs, 22 tRNA and 2 *rRNA* genes is detailed in Table 2.

**Table 2:** Mitochondrial genome characteristics of *Pseudolebinthus lunipterus*.

Gene	Location	Length (bp)	Strand	Start codon	Stop codon	Anticodon sequence
tRNA-Ile	1–63	63	+			GAT
tRNA-Gln	61–129	69	−			TTG
tRNA-Met	147–215	69	+			CAT
*ND2*	217–1,233	1,017	+	ATT	TAA	
tRNA-Trp	1,232–1,296	65	+			TCA
tRNA-Cys	1,289–1,351	63	−			GCA
tRNA-Tyr	1,367–1,430	64	−			GTA
*COX1*	1,424–2,971	1,548	+	ATC	TAA	
tRNA-Leu^2^	2,966–3,030	65	+			TAA
*COX2*	3,031–3,706	676	+	ATG	TAA^1^	
tRNA-Lys	3,706–3,775	70	+			CTT
tRNA-Asp	3,776–3,843	68	+			GTC
*ATP8*	3,845–4,003	159	+	ATT	TAG	
*ATP6*	3,997–4,677	678	+	ATG	TAA	
*COX3*	4,681–5,467	787	+	ATG	TAA^1^	
tRNA-Gly	5,467–5,530	64	+			TCC
*ND3*	5,532–5,885	354	+	ATA	TAA	
tRNA-Ala	5,886–5,949	64	+			TGC
tRNA-Arg	5,950–6,012	63	+			TCG
tRNA-Glu	6,007–6,070	64	−			TTC
tRNA-Ser^1^	6,071–6,137	67	−			GCT
tRNA-Asn	6,138–6,203	66	−			GTT
tRNA-Phe	6,211–6,278	68	−			GAA
*ND5*	6,251–7,927	1,677	−	CAT	TTA	
tRNA-His	8,002–8,064	63	−			GTG
*ND4*	8,066–9,409	1,342	−	CAT	TTA	
*ND4L*	9,403–9,687	285	−	CAT	TTA	
tRNA-Thr	9,704–9,767	64	+			TGT
tRNA-Pro	9,768–9,833	66	−			TGG
*ND6*	9,837–10,361	525	+	ATT	TAA	
*CYTB*	10,361–11,500	1,140	+	ATG	TAA	
tRNA-Ser^2^	11,499–11,563	65	+			TGA
*ND1*	11,564–12,518	952	−	TAT	TAA^1^	
tRNA-Leu^1^	12,521–12,587	67	−			TAG
*Large subunit rRNA*	12,599–13,895	1,297	−			
tRNA-Val	13,877–13,944	68	−			TAC
*Small subunit rRNA*	13,944–14,733	790	−			
A+T rich region	14,734–16,075	1,342				

**Note:**

Direction of transcription: +, forward; −, reverse. Exponent numerals in gene column differentiate each of the two leucine- and serine-specifying tRNAs (Leu1 and Leu2, Ser1 and Ser2). 1TAA stop codon is completed by the addition of 3′A residues to mRNA.

### Phylogenetic relationships

**Figure 3:**
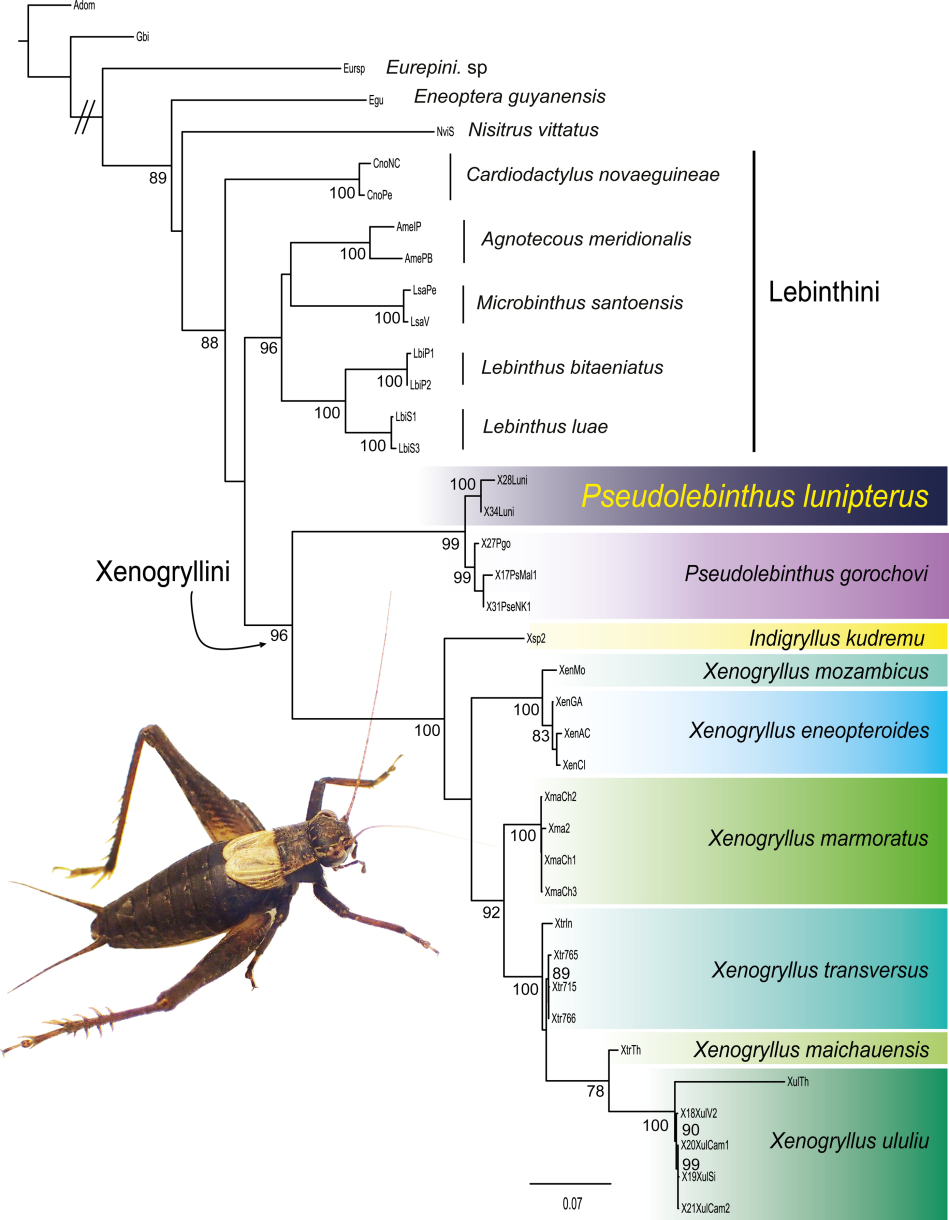
Phylogenetic position of *Pseudolebinthus lunipterus* sp. nov. Maximum likelihood tree of Xenogryllini tribe based on the concatenated dataset of eight genetic markers. ML bootstrap (BS) values higher than 75% are indicated for each node on the left. Clades corresponding to species are shaded with a color scale. Represented species: male of *Pseudolebinthus lunipterus* sp. nov. Photo Tony Robillard.

The alignment of all eight markers consists of 3,684 aligned base pairs (bp) for 34 terminals: 416 bp for 12S, 521 bp for 16S, 707 bp for CO1, 335 bp for CO2, 346 bp for Cytb, 328 bp for H3, 652 bp for 18S and 379 bp for 28S. The ML phylogenetic tree inferred from this data is shown in Fig. 3.

Most nodes of the tree have high bootstrap supports and are consistent with the recent study of Jaiswara, Dong & Robillard (2019). All species represented by two or more terminals are found monophyletic. The subfamily Eneopterinae and the tribe Xenogryllini are recovered as monophyletic with high support values (BS of 100% for Eneopterinae, 99% for Xenogryllini). The relationship of tribes Lebinthini and Xenogryllini are also supported, although the Lebinthini are not found monophyletic. The new species occurs as the sister species of *P. gorochovi* with high support (BS of 100%). Branches between the two species crown groups are short, suggesting that the new species should be considered as a member of the genus *Pseudolebinthus*.

**Taxonomy**

**Insecta** Linnaeus, 1758

**Orthoptera** Olivier, 1789

**Gryllidae** Laicharting, 1781

**Eneopterinae** Saussure, 1874

**Xenogryllini** Robillard, 2004

**Genus *Pseudolebinthus*** Robillard, 2006

Type species: *Pseudolebinthus africanus* Robillard, 2006

***P. lunipterus* sp. nov.**

(Figs. 1–11)

**Type material**

Holotype male, MALAWI. N. Malawi, Mt. Uzumara, 6,500 ft. R.J. Murphy col. 2.i.2001 (MNHN-EO-ENSIF10715). Allotype female, same information as holotype (MNHN-EO-ENSIF10716). Paratypes (9♂, 6♀): MALAWI. Same information as holotype, 1♂, #233RJM (MNHN-EO-ENSIF10717). N. Malawi, Mount Uzumara (MAL1), S10°52′19,3″ E34°07′44,7″, 1,941 m (MAL1), 2-4.x.2018, nuit (night), plante, élevage F0 (collected as juveniles on plant, reared to final moult in captivity), T. Robillard, K. Salazar & R.J. Murphy: 3♂, molecular sample X34, X45, X28 (MNHN-EO-ENSIF10718-10720); 2♂, video of mating behavior (♂#3, ♂#4) (MNHN-EO-ENSIF10721-10722); 3♂ (MNHN-EO-ENSIF10721-10722); 2♀, video of mating behavior (♀#1, ♀#3) (MNHN-EO-ENSIF10723-10724); 4♀ (MNHN-EO-ENSIF10725-10728).

**Additional material examined.** MALAWI. N. Malawi, Mount Uzumara (MAL1), S10°52′19,3″ E34°07′44,7″, 1,941 m (MAL1), 2019, élevage F1, T. Robillard: 1♂, 1♀, 3 juveniles (MNHN).

**Type locality.** North Malawi, Mount Uzumara, S10°52′19,3″ E34°07′44,7″, 1,941 m.

**Distribution.** The species is only known from the type locality in Northern Malawi (Fig. 1C).

**Etymology.** The species name refers to the whitish wings, rounded in males and crescent-shaped in females, which look like tiny moons on the back of the dark body of these crickets when encountered at night.

**Diagnosis**

**Figure 4:**
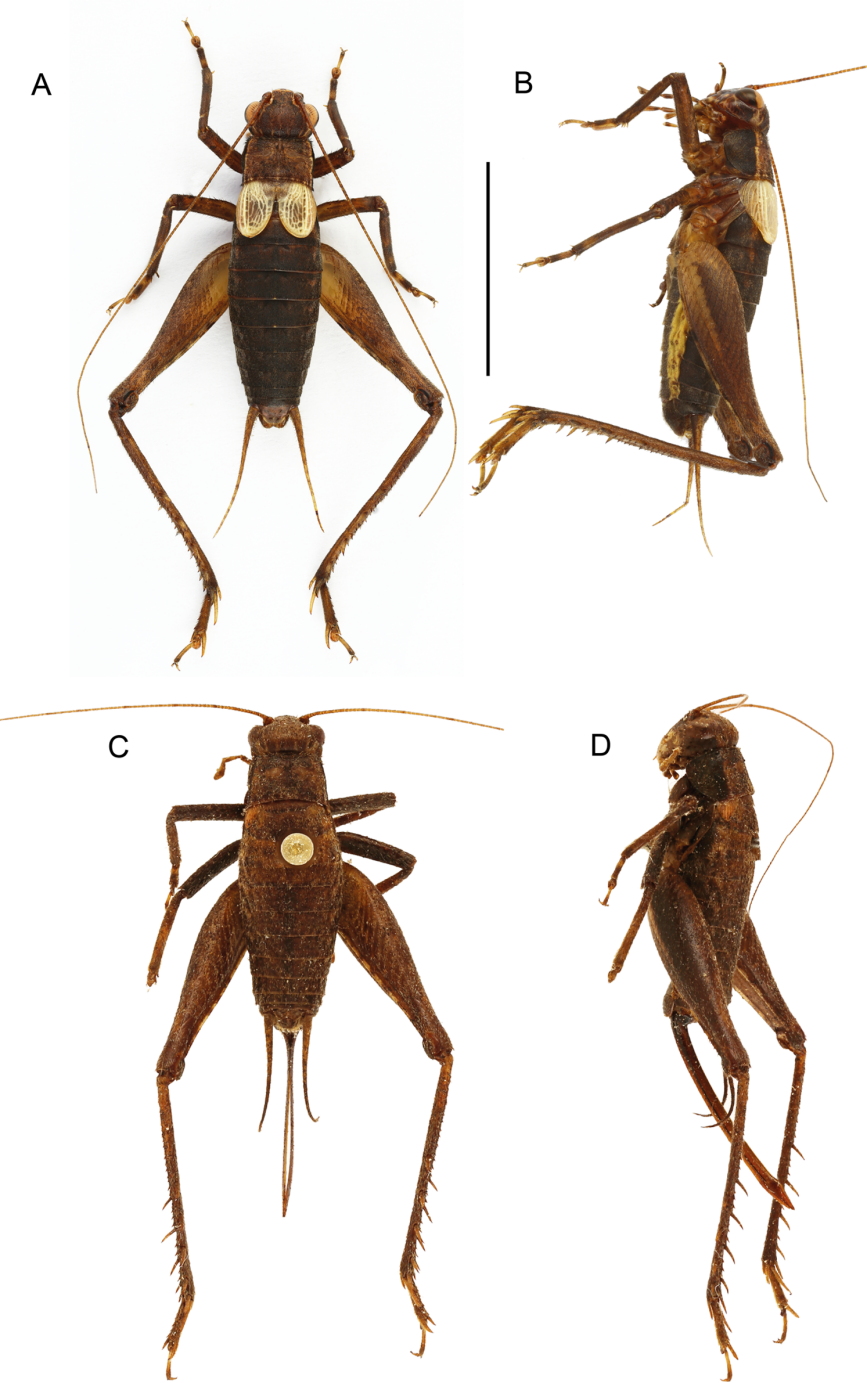
Male and female of *Pseudolebinthus lunipterus* sp. nov. (A and B) Male and (C and D) female in dorsal (A and C) and lateral (B and D) views. Scale bar = 1 cm. Photos (A and B) Karen Salazar. (C and D) Simon Poulain.

**Figure 5:**
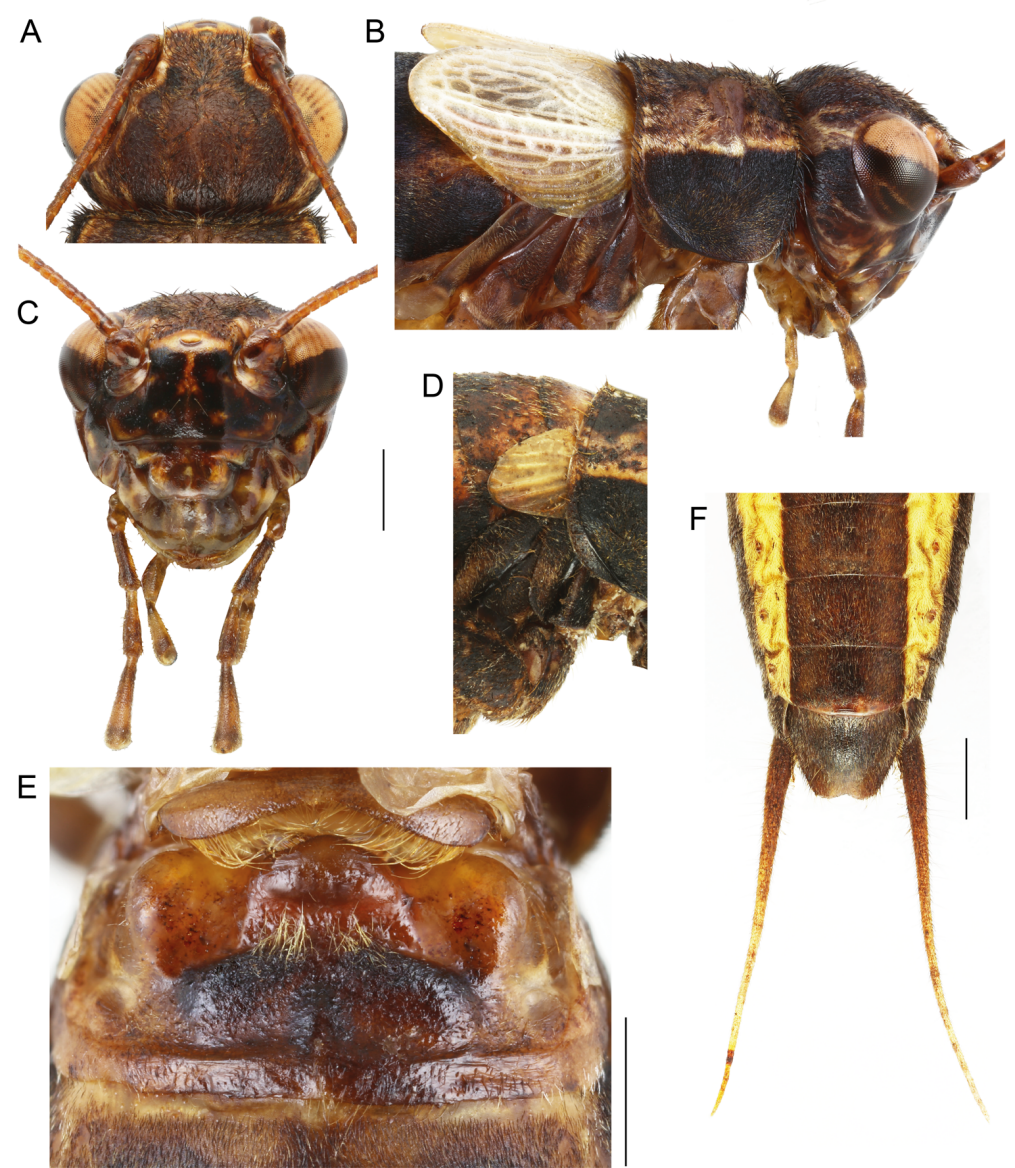
Morphology of *Pseudolebinthus lunipterus* sp. nov. (A–C, E and F) Male and (D) female; (A–C) head in dorsal (A) and facial (C) views; (B) head, pronotum and wings in lateral view; (D) lateral view of wings; (E) metanotal glands; (F) ventral face of tip of abdomen, subgenital plate and cerci. Scale bars = 1 mm. Photos Karen Salazar.

**Figure 6:**
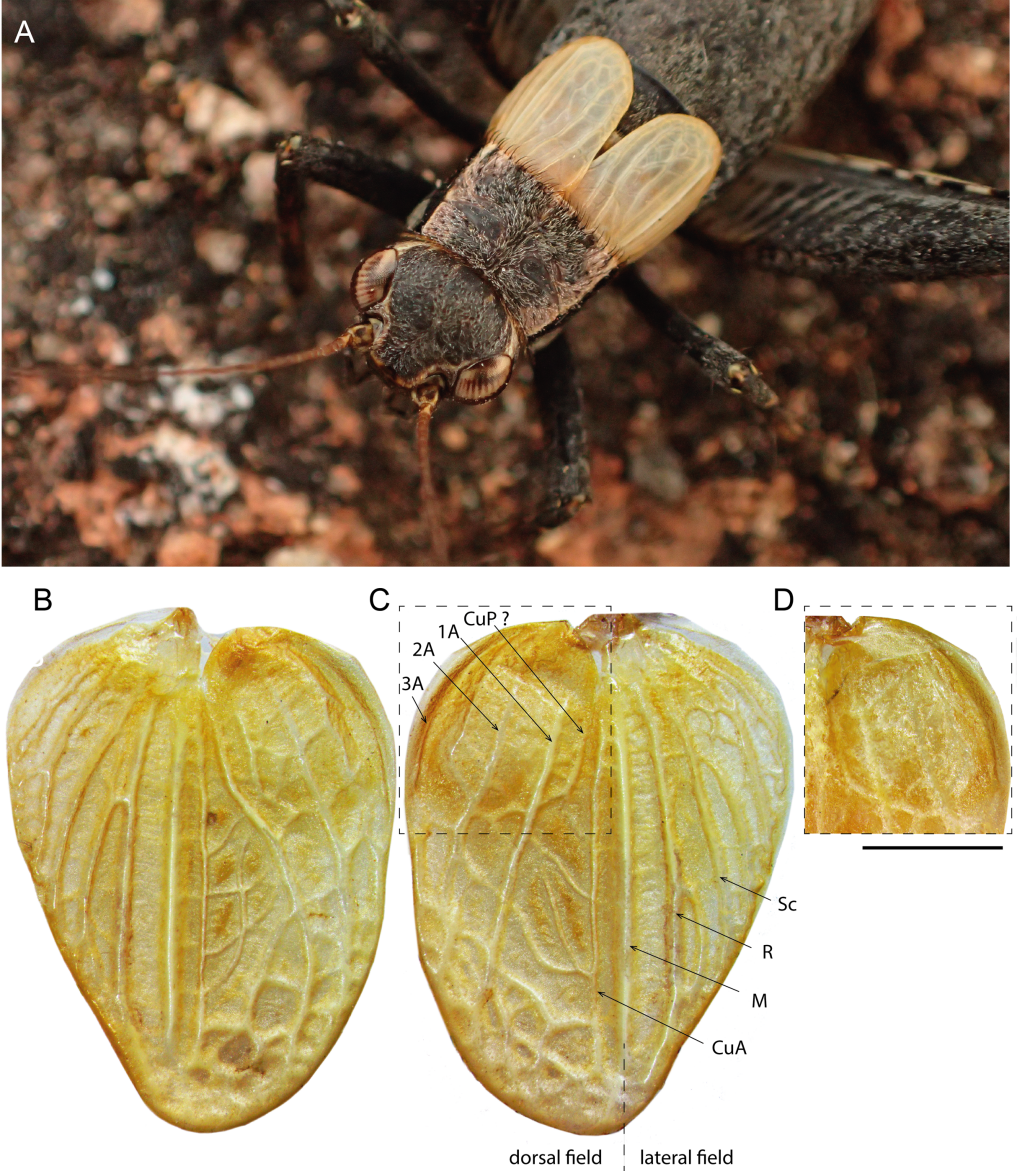
Male forewings and venation of *Pseudolebinthus lunipterus* sp. nov. (A) Male habitus showing FW coloration in their natural context; (B–D) detailed view of left (B) and right (C) FW in dorsal view; (D) anterior part of right FW in ventral view. Abbreviations: see “Material and Methods”. Scale bar = 1 mm. Photos Tony Robillard.

**Figure 7:**
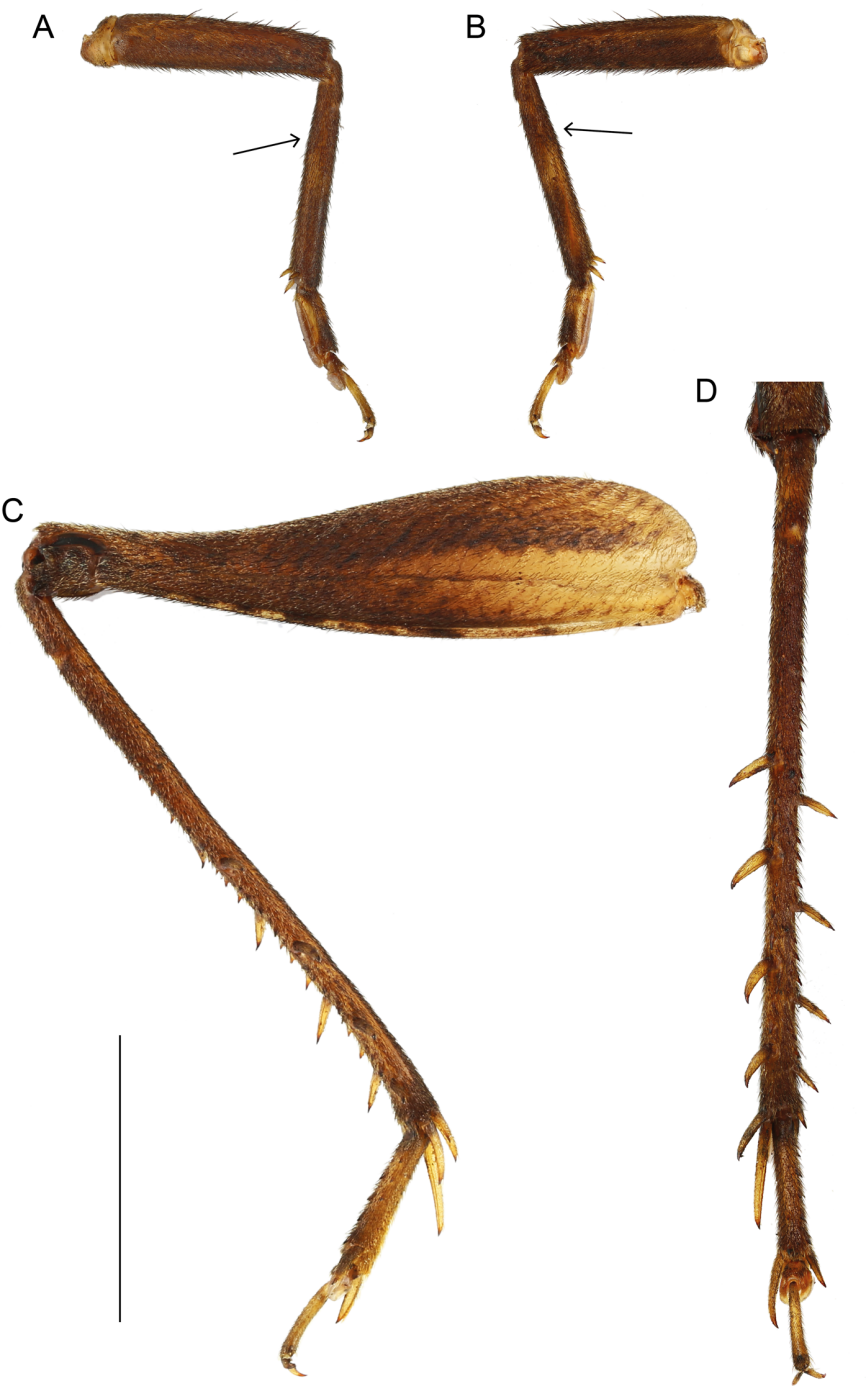
Legs of *Pseudolebinthus lunipterus* sp. nov. (A and B) Posterior and anterior views of fore leg showing no trace of tympanum on upper part of tibia (see arrows); (C) lateral external view of hind leg; (D) detail of hind tibia in dorsal view. Scale bar = 5 mm. Photos Karen Salazar.

**Figure 8:**
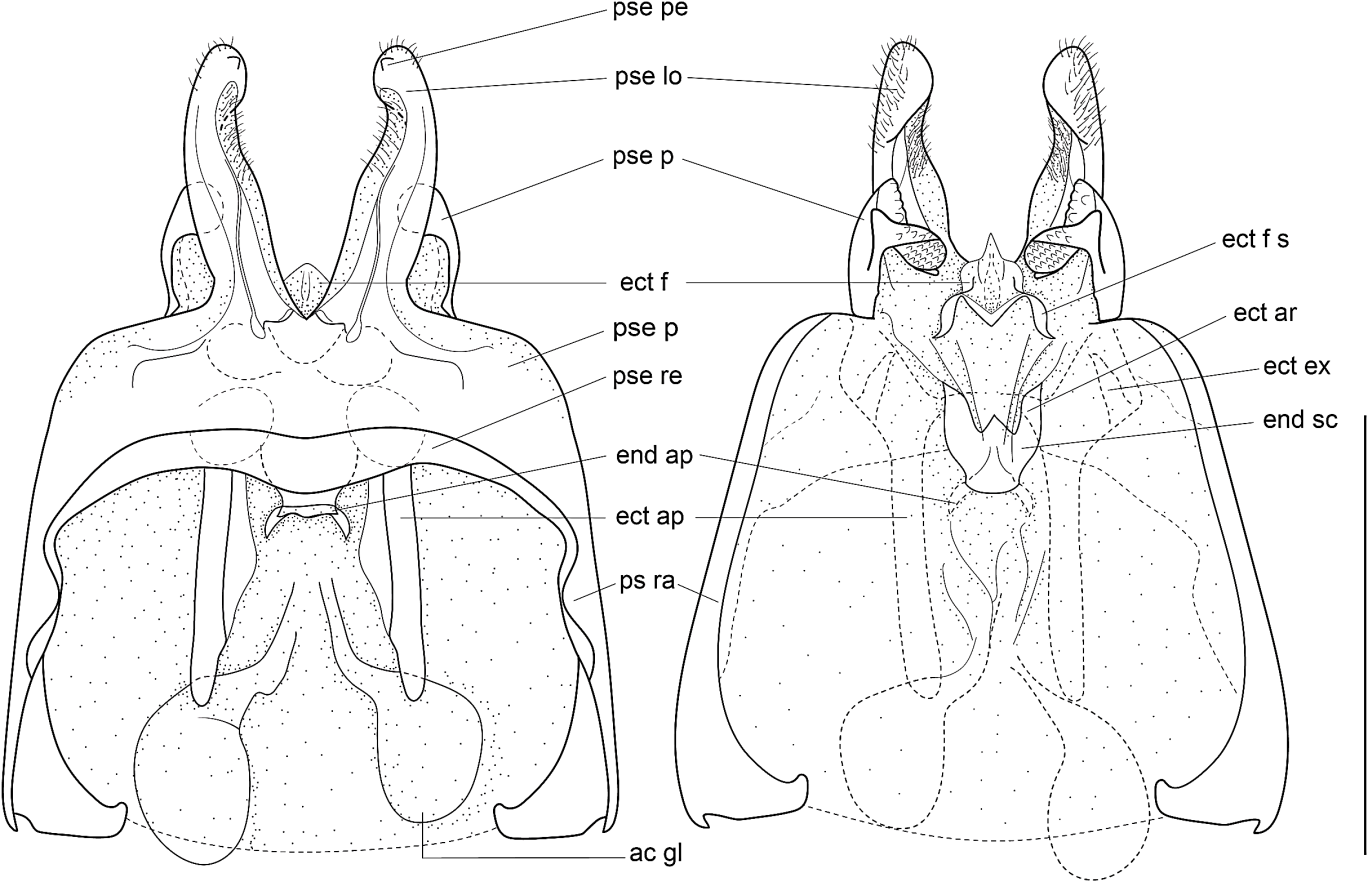
Male genitalia of *Pseudolebinthus lunipterus* sp. nov. (A) Dorsal and (B) ventral views; dotted parts represent membranous areas. Abbreviations: see “Material and Methods” section. Scale bar = 1 mm. Drawing Karen Salazar & Tony Robillard.

**Figure 9:**
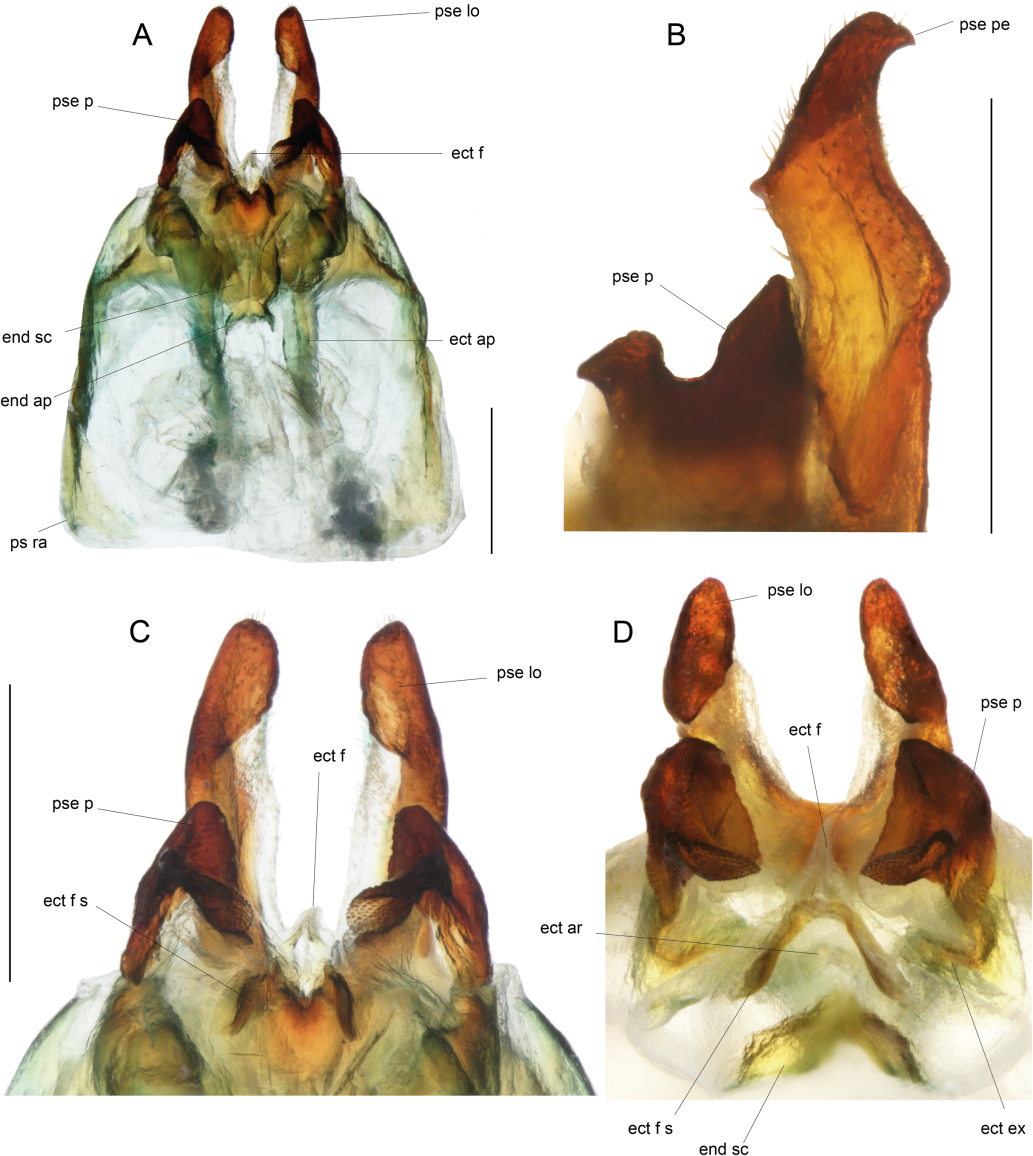
Details views of male genitalia. (A) Complete ventral view; (B) lateral view of pseudepiphallic lophi; (C and D) pseudepiphallic lophi and parameres, and ectophallic fold in ventral (C) and posterior views. Scale bar = 0.5 mm. Photos Karen Salazar.

**Figure 10:**
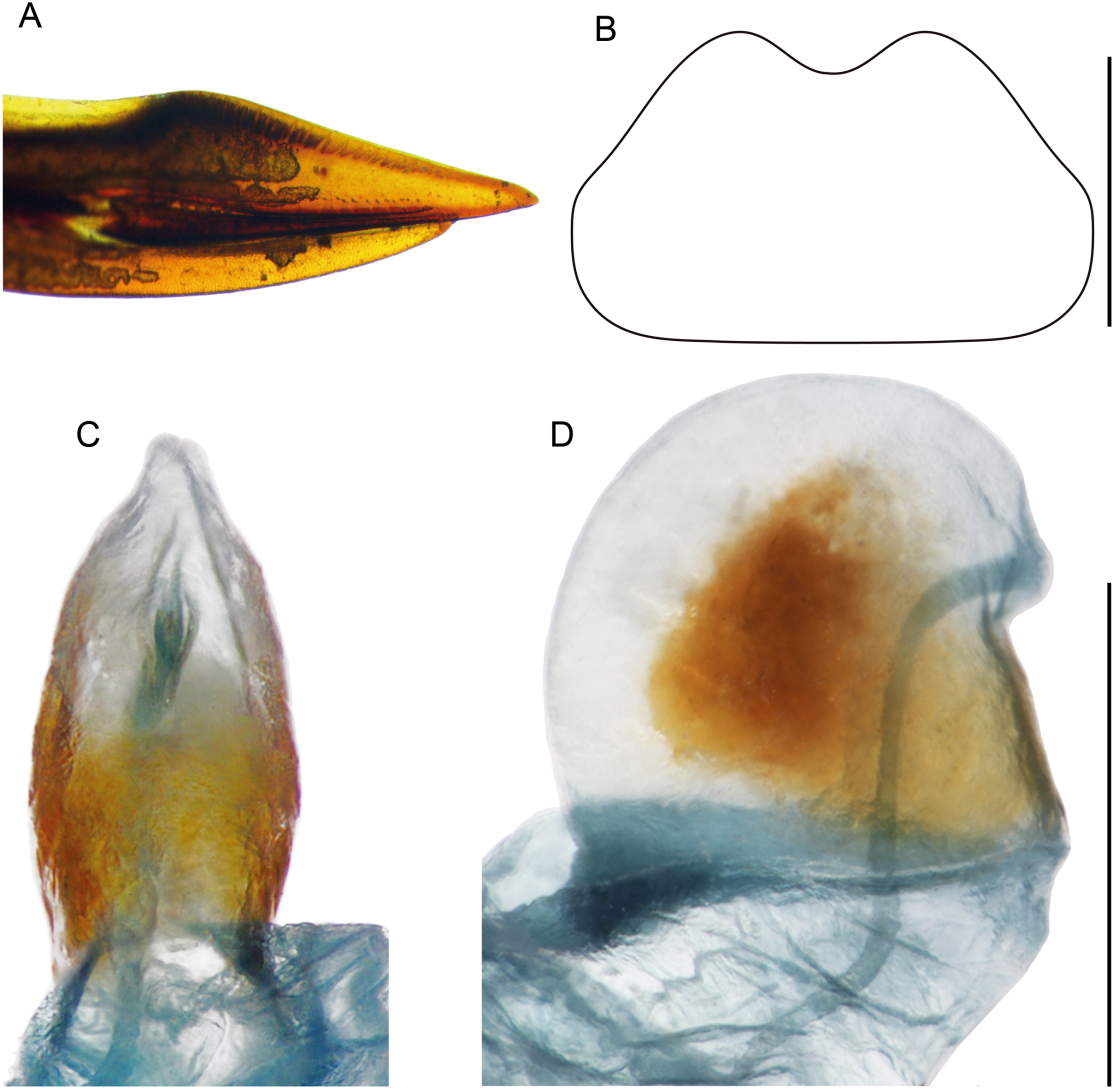
Female structures of *Pseudolebinthus lunipterus* sp. nov. (A) Apex of ovipositor in lateral view; (B) shape of subgenital plate; (C and D) copulatory papilla in ventral (C) and lateral (D) views. Scale bar = 0.5 mm. Photos and drawing Karen Salazar.

Size small, mostly dark brown with pale wings (Figs. 1, 3 and 4). Among Eneopterinae genera, the new species presents the characteristics of *Pseudolebinthus*: large lateral eyes (Figs. 5A–5C); brachypterous FWs barely reaching quarter of abdomen length in males (Figs. 4 and 6), shorter in females where it forms pale narrow crescents (Figs. 4 and 5D); male genitalia with long sclerotized lophi, close to that of *P. gorochovi* (Figs. 8 and 9); female ovipositor little differentiated but less pointed and thicker than in *P. gorochovi* (Fig. 10A). The new species is characterized by complete absence of tympana (unique feature among eneopterines) (Figs. 7A and 7B), absence of stridulatory apparatus on male FWs (Fig. 6), abdomen ventrally yellow with a wide black stripe (Fig. 5F), thick and short female ovipositor (Fig. 4D), and differences in male genitalia, including shape of pseudepiphallic parameres, shape of sclerite in ectophallic fold and endophallic apodeme with anterior lateral expansions.

**Description**

In addition to the characters of the genus: body mostly dark brown with pale wings (Figs. 1, 3 and 4). Head rounded dorsally, with five black bands separated by thin yellow lines, including two thin bands posterior to eyes and three wider median bands, sometimes partly faded in median area (Figs. 5A–5C). Fastigium black, setose, with thin yellow margins, its apex with a wide yellow band surrounding median ocellus. Eyes occupying ca. 40% of total head width in dorsal view. Ocelli forming a wide triangle. Scapes short, wider than long, black with a yellow spot on upper inner side; rest of antennae brown. Face triangular (Fig. 5C), almost entirely black except: a median vertical line connected to the yellow band at fastigium apex, two yellow spots above epistomal suture, a yellow spot below each eye; mouthparts black, mottled with yellow. Lateral part of head entirely black (Fig. 5B). Pronotum dark brown dorsally, its lateral margins thinly underlined with yellow (Figs. 5B and 6A); lateral field black (Fig. 5B). TI with no tympana (Figs. 7A and 7B). FWs very short in both sexes (Fig. 4), hind wings absent. Fore and median legs with black femora, dark brown tibiae (Fig. 4); base of tarsomeres yellow, apex darker. FIII external face bicolor: dorsal half black, ventral half yellow; knees black (Fig. 7C); TIII dark brown; tarsomere III yellow basally, apex dark brown. Abdomen rather long and fusiform (Fig. 4), yellow ventrally with a wide black stripe, including subgenital plate (Fig. 5F). Subgenital plate slightly indented apically in both sexes (Figs. 5F and 10B).

**Males.** FWs short, reaching one quarter of abdomen length (Figs. 4A, 4B and 6); without stridulatory apparatus; veins and cells soft, without pigmentation (whitish in the living, turning yellow when drying). Dorsal field (Figs. 6B and 6C) with two main strong longitudinal veins corresponding to veins 1A and 2A; CuA weak, with two apical expansions delimiting possible cell alignment of apical field; and one longitudinal vein along inner margin (3A?). Area delimited by 1A and CuA slightly widened posteriorly, sometimes with a faint anterior vein possibly corresponding to vein CuP; area with variable transverse veins in posterior region, possibly corresponding to diagonal vein or posterior limit of cells of mirror area in crickets having a stridulatory apparatus. Ventral face of 1A without trace of stridulatory teeth (Fig. 6D). Lateral field narrow (Figs. 6B and 6C), with three main longitudinal veins, including R, Sc and M, the latter separating dorsal and lateral fields; one more ventral vein sometimes near FW base. Hind wings vestigial. Metanotum with glandular structures (Fig. 5E); gland morphology close to that of other *Pseudolebinthus*, with a bunch of long setae on basal margin, a wide median process on scutum, and basal edge of scutellum raised medially and carrying a bunch of setae orientated anteriorly; posterior part of mesonotum setose and extended posteriorly, covering anterior part of metanotal scutum. Male subgenital plate elongate (Fig. 5F).

Male genitalia (Figs. 8 and 9). Pseudepiphallic sclerite as long as rami, widened laterally near base of rami. Pseudepiphallic lophi thin and parallel, longer than in *P. gorochovi*, twisted ventro-apically; their apex with a small dorsal expansion. Pseudepiphallic parameres dorsal lobe triangular, longer than ventral lobe; ventral lobe oriented anteriorly forming an apical fold. Ectophallic fold with a strong ventro-lateral sclerotization forming a transversal bridge (Figs. 8B, 9C and 9D) slightly extended posteriorly within pointed membranous apex. Ectophallic apodemes long and parallel. Endophallic sclerite with a small median area, with wide lateral arms; endophallic apodeme including two small dorso-anterior arms at anterior apex of endophallic sclerite and a narrow apical transverse crest extended laterally, underlying arms of endophallic sclerite.

**Figure 11:**
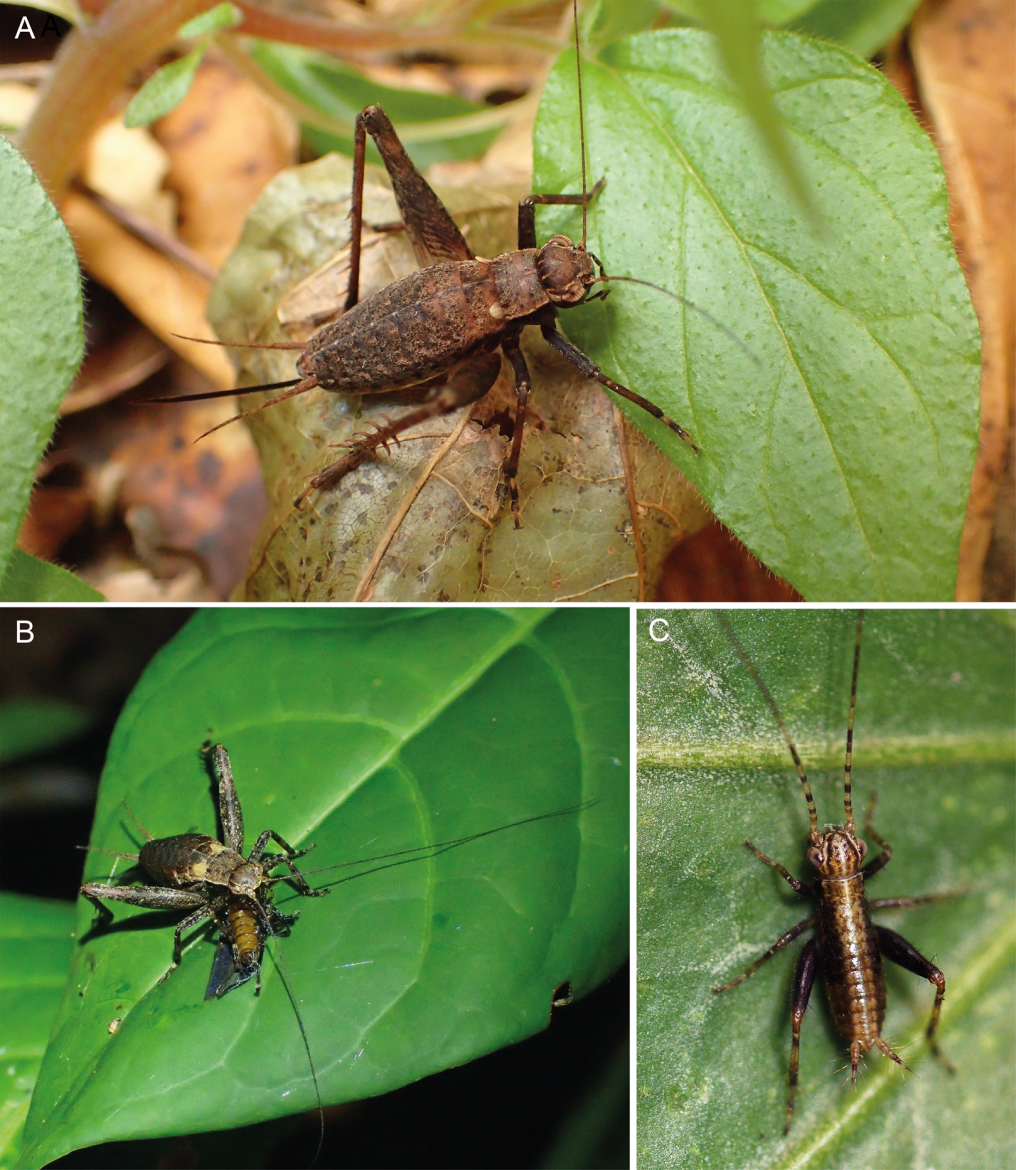
Live photos of *Pseudolebinthus lunipterus* sp. nov. (A) Female on vegetation; (B) subadult male eating a dead insect on a leaf at night; (C) first instar juvenile. Photos (A and B) Tony Robillard and (C) Karen Salazar.

**Females** (Figs. 4C, 4D, 5D, 10 and 11A). FWs forming very small lateral whitish leaflets (Fig. 5D), not overlapping and not reaching posterior margin of first tergite (Figs. 4C, 4D and 5D). Ovipositor short and thick, shorter than FIII (Figs. 4C and 4D), its apex little differentiated, pointed and not denticulate dorsally (Fig. 10A). Female copulatory papilla flattened laterally, mostly sclerotized, its apex membranous folded ventrally (Figs. 10C and 10D).

**Juveniles.** First instars body cylindrical (Fig. 11C), mostly dark brown with a light dorsal stripe; antennae brown with yellow rings. Later instars with similar coloration pattern as adults, usually lighter brown. Last instar with characteristic whitish wing buds, similar to wing coloration in adults (Fig. 11B).

**Habitat and life history.** *Pseudolebinthus lunipterus* lives on low vegetation in herbaceous areas near forest hedge or in open areas along trails in forest (Figs. 1A and 1B). Adults and juveniles have been found active at night on top of vegetation, but can also be found lower within vegetation during the day. Remarkably, the species lives in syntopy with *P. gorochovi* in the type locality, where adults and juveniles of both species are quite abundant. One juvenile specimen of *P. lunipterus* has been observed eating a dead insect on a low leaf on vegetation (Fig. 11B).

Females maintained in controlled laboratory conditions (20–22 °C, 14–10 day–night cycle) with a single male produced 46–50 offspring (*n* = 2) during their life; first hatchings started 42–49 days after first mating and occurred on a period of 35–66 days.

**Measurements** (in mm). See Table 3.

**Key to *Pseudolebinthus* species modified from Jaiswara, Dong & Robillard (2018)**

1. TI each with one pair of tympana, male FWs longer than one quarter of abdomen length, with a stridulatory apparatus2

– TI without tympana, male FWs about one quarter of abdomen length, without stridulatory apparatus***P. lunipterus* sp. nov.**

2. Dorsal margin of eye with small ommatidia narrow. Male FW venation: mirror small, hardly distinct from surrounding cells. Male genitalia: pseudepiphallic lophi long and thin*P. africanus* Robillard, 2006

– Dorsal margin of eye with small ommatidia wider. Male FW venation: mirror larger, wider than long, well differentiated. Male genitalia: pseudepiphallic lophi shorter3

3. Coloration little contrasted; FIII homogeneously brown on external face. Male FW venation: c1 and c2 cells sub-equal, thin. Vein Sc with four projections along its length. Male genitalia: apex of pseudepiphallic lophi bilobate*P. whellani* Robillard, 2006

– Coloration more contrasted; FIII external face bicolor, dorsal half dark brown, ventral half yellow. Male FW venation: c1 cell wide, c2 twice wider than c1, square and prolonging shape of miror. Vein Sc with two projections along its length. Male genitalia: apex of pseudepiphallic lophi twisted ventrally, not bilobate*P. gorochovi* Robillard, 2018

**Table 3:** Measurements of *Pseudolebinthus lunipterus* sp. nov. Measurements in mm except spine numbers; abbreviations, see “Materials and Methods”.

	BL	PronL	PronW	FWL	FWW	FIIIL	FIIIW
Holotype male	13.6	1.9	3.1	2.8	1.6	9.1	2.5
Males (*n* = 5)	13.5–14	1.8–2.1	2.9–3.3	2.8–3.5	1.6–1.9	9.1–11.2	2.5–3
(Male mean)	(13.7)	(1.9)	(3.1)	(3.1)	(1.8)	(10.5)	(2.8)
Female Allotype	15.1	2.2	3.5	1.1	–	11.8	3.2
Females (*n* = 5)	15.1–16.9	2.2–2.5	3.2–3.8	0.5–1.4	–	11–12.1	3.2–3.5
(Female mean)	(15.7)	(2.3)	(3.6)	(0.9)	–	(11.5)	(3.3)

	TIIIL	TIIIs				TaIIIs			Ols
		Ias	Ibs	Oas	Obs	Ids	Ods	OL	
Holotype male	9	7	7	10	8	1	3	2	–
Males (*n* = 5)	9–11	6–7	7–11	8–11	8–12	0–1	3–5	2–3	–
(Male mean)	(9.8)	(7)	(9)	(10)	(10)	(1)	(4)	(3)	–
Female Allotype	10.5	5	8	11	8	0	4	3	10.8
Females (*n* = 5)	10.5–12.1	5–8	7–10	9–12	8–11	0	3–5	3	9.3–10.8
(Female mean)	(11.1)	(6)	(9)	(11)	(9)	(0)	(4)	(3)	(10.1)

## Discussion

### Multiple losses of acoustic communication

In this article we described the species *P. lunipterus* sp. nov., a new eneopterine cricket from Northern Malawi being both mute and deaf. This new species is the first reported case showing complete absence of stridulatory apparatus (no stridulatory file, harp and mirror) in this cricket clade, associated with absence of tympana on both sides of fore tibiae.

Our taxonomic study suggests that *P. lunipterus* belongs to the tribe Xenogryllini despites all its special morphological features. The phylogenetic analysis shows that it is closely related with at least one other species of the genus *Pseudolebinthus* (Fig. 3). This phylogenetic position has two interesting consequences:

First, this is the first case of muteness documented in the clade Xenogryllini. Two other cases of loss of acoustic communication were previously reported in eneopterines (Table 4): one occurred in the tribe Nisitrini and concerns the apterous species of the genus *Paranisitra*, which has diverged from its sister genus *Nisitrus* ca. 55 Ma, before diversifying in the Philippines after 12.5 Myrs (Vicente et al., 2017; Baroga-Barbecho et al., 2019). The second other mute lineage is the genus *Swezwilderia* in the tribe Lebinthini; species of *Swezwilderia* possess long wings, but lack stridulatory structures. This genus has diverged from its sister group ca. 48.4 Ma, and has diversified in Fiji and Samoa after 21.6 Myrs (Vicente et al., 2017). In addition to their phylogenetic independence, these three losses of calling abilities are structurally different, one occurring through the loss of complete wings and the two others consisting of losses of stridulatory structures either on fully formed wings (*Swezwilderia*) or on reduced wings (*P. lunipterus*). These different combinations of traits support the hypothesis that these three losses of acoustic communication are convergent in Eneopterinae. The phylogenetic context of each loss may explain these different configurations in relation with two other functions of the wings of insects: flight and protection. In *Swezwilderia*, the wings might have been retained in association with keeping flying capacities, while short wings might have been necessary in *P. lunipterus* to protect the metanotal glands that are shared among all the Xenogryllini species (Jaiswara, Dong & Robillard, 2018, 2019; Jaiswara et al., 2019). Interestingly, the short wings in the new species are shorter than that of other *Pseudolebinthus* species, but remain just long enough to cover the glands beneath (Figs. 5B, 5E and 6). In contrast, *Paranisitra* is apterous but has also lost the metanotal glands while diverging from *Nisitrus*.

**Table 4:** Traits linked to absence of acoustic communication across Eneopterinae subfamily.

Tribe	Taxon	Wings	Flight	Metanotal glands	Stridulatory structures	Inner tympanum	Outer tympanum	Hearing
Xenogryllini	*P. lunipterus*	Short	No	Present	Absent	Absent	Absent	Absent
		*Pseudolebinthus* (other species)	Short	No	Present	Present	Present	Present
		*Xenogryllus*	Long	Yes	Present	Present	Present	Present
Nisitrini	*Paranisitra*	Absent	No	Absent	Absent	Present	Present	Present
		*Nisitrus*	Long	Yes	Present	Present	Present	Present
Lebinthini	*Swezwilderia*	Long	Yes	Absent	Absent	Present	Present	Present
		Lebinthini (other genera)	Long/short	Yes/no	Absent	Present	Present	Present
Eneopterini	No mute taxon	Long	Yes	Present	Present	Present	Present	Present
Eurepini	No mute taxon	Long/short	Yes/no	Variable	Present	Present	Absent	Present

**Note:**

Mute taxa discussed in the text are highlighted in gray.

The second interesting observation about the phylogenetic position of *P. lunipterus* is that this is the only mute species occurring within a genus, while others mute cases concern clear-cut genera which show strong divergence from their sister lineages. Even if genera do not represent evolutionary units, it is interesting to notice that *P. lunipterus* and *P. gorochovi* are separated by very short branches in the phylogenetic tree (Fig. 3). A molecular dating analysis of *Pseudolebinthus* will be necessary with a better taxonomic sampling, but this situation suggests that the loss of acoustic communication in *P. lunipterus* is likely a recent event, which is recalling the loss of sound production structures occuring convergently and very rapidly within some populations of *T. oceanicus* as a result to strong selective pressures by a parasitoid fly (Zuk, Rotenberry & Tinghitella, 2006; Pascoal et al., 2014). Analogous selective pressures might be responsible for the loss of sound production in *P. lunipterus*.

The most unique feature of *P. lunipterus* at the scale of the subfamily is the deafness of the species. Other cases of deaf crickets have been documented in other clades, but this is the only case known in eneopterines. In many mute lineages of crickets, auditory tympana are retained after the stridulatory mechanism is lost (Otte, 1992). In species that are still able to fly, but in which males have lost the stridulum (such as in species of *Swezwilderia*), the tympanum is usually retained, which is supposed to be linked with avoidance of bat predation in flight (Otte & Alexander, 1983; Otte, Alexander & Cade, 1987). Species becoming both mute and deaf, such as *P. lunipterus*, are less common. This combination of traits might be explained by predator avoidance selecting for mute crickets in lineages having ancestrally lost their flying capacities (all *Pseudolebinthus*). In such cases, maintaining tympana might not be necessary. Interestingly, this hypothesis does not hold with the case of *Paranisitra*, which lost the wings while retaining hearing capacities (or at least external organs, since the hearing capacities of *Paranisitra* have never been evaluated).

### Generic allocation

The morphological study of the new species shows that it shares all the characteristics of the genus *Pseudolebinthus* in terms of general morphology, body size and main features of male genitalia. On the other hand, the new species differs by important characters such as FW length, absence of stridulatory file and tympana, and by the shape of female copulatory papilla. Such differences suggest that the new species might have been considered as an easily recognized new genus close to *Pseudolebinthus*. This hypothesis has been tested using the molecular data and the phylogenetic relationships. Although it is too early to conclude that the new species is nested within *Pseudolebinthus* (only one previously described species was successfully sequenced here), our results clearly show that the new species is very close to *P. gorochovi*, and the short stem branches in the phylogeny leading to the new species and *P. gorochovi* strongly support the hypothesis that the new species should be considered as a particular species of *Pseudolebinthus*.

## Conclusion: Crickets of Malawi

The diversity of crickets in Eastern Africa in general, and Malawi in particular, has been underestimated, understudied and undersampled. This is at least the case for the members of the tribe Xenogryllini which were recently revised (Jaiswara, Dong & Robillard, 2018, 2019; Jaiswara et al., 2019). Despite the large amount of data considered in these systematic studies (several hundreds of specimens studied across the study of the largest natural history museum collections), they gathered very little information about the species of *Pseudolebinthus*, known by a few specimens each.

A single recent field trip in Malawi allowed us to re-discover two of the previously described species of the genus, which are in fact common species, and it allowed documenting the acoustic features of their calling songs and their ecology (T. Robillard et al., 2020, in prep.). Interestingly, these findings allowed us to discover *P. lunipterus*, a completely different new species belonging to the Xenogryllini lineage, but with strikingly new morphological features. This finding reveals that more taxa probably remain unrecorded in the whole Eastern African region, as suggested by the large amount of new species and genera recently discovered in this region for other clades of orthopteran insects (Hemp et al., 2018; Hemp & Heller, 2019). More taxonomic surveys with appropriate collecting methods in regions where there is zero record about these crickets, such as other regions of Malawi, but also Zimbabwe, Zambia, Western Mozambique and Northern South Africa, are thus necessary to explore this part of African biodiversity.

## Supplemental Information

10.7717/peerj.8204/supp-1 Supplemental Information 1 Sequences submitted to GenBank. Accession numbers processed by GenBank after sumission. This supplementary material will be deleted when accession numbers will be made available.

## Additional Information and Declarations

### Field Study Permissions

The following information was supplied relating to field study approvals (i.e., approving body and any reference numbers): The Forestry Research Institute of Malawi (FRIM) granted a collection and export permit for this study (EAD-12-07-087-18-20a).

### DNA Deposition

The following information was supplied regarding the deposition of DNA sequences: Sequence data are available at NCBI under GenBank Accession Numbers listed in Table 1.

### Data Availability

The following information was supplied regarding data availability: Specimens are deposited in the MNHN specimen data base (https://science.mnhn.fr/institution/mnhn/search?lang=fr_FR) under number given in the section “type material” as numbers “MNHN-E0-ENSIF***”.

### New Species Registration

The following information was supplied regarding the registration of a newly described species: Publication LSID: urn:lsid:zoobank.org:pub:DA17C29A-B265-4819-A9E6-55FCB137E3F0. *P. lunipterus* sp. nov. LSID: urn:lsid:zoobank.org:act:1DFFBC9E-6C42-4DDB-9EB4-87CB9E6A6756
